# Supplementary material for: LI-Detector: a Method for Curating Ordered Gene-Replacement Libraries
Source: Microbiol Spectr. 2022 Jul 20;10(4):e00833-22. doi: 10.1128/spectrum.00833-22 (PMC9431181; doi:10.1128/spectrum.00833-22)
Supplement: Supplemental file 1 — Supplemental material. Download spectrum.00833-22-s0001.pdf, PDF file, 0.5 MB [file spectrum.00833-22-s0001.pdf]

Supplementary Information

Primers that did not map to the BW25113 reference genome:

```
ycgR primer:  TTTGAGCTTTTTCTGCTACCGTTTGCCCATCAATCAGCGTCGCTCCGGCG
              |||||||||||||||||||||||||||||||||||||||||||||||
genome:       TTTGAGCTTTTTCTGCTACCGTTTGCCCATCAATCAGCGTCGCTCCGCGT
```

```
argI primer:  GATGG-TTTCC-G-CATCTT-ATAGCGATCAG--ATTATTTACTGAGCGTCGCGAC
                ||   ||||| | |||||| |||||||| ||  ||||||||||||||||||||||
genome:        TTGTATTTCCCGGCATCTTTATAGCGAT-AGCAATTATTTACTGAGCGTCGCGAC
```

```
lacI primer:  TGAGCTAACTCACATTAATTGCGTTGCGCTCACTGCCCGCTTTCCAGTCG
                                  ||||||||||||||||||||||||||||||
genome:      GCAGATGCGGTCCTCAATCGCGCGTTGCGCTCACTGCCCGCTTTCCAGTCG
```

```
=30 nt homology
```

eutA primer up: CCAAAAGAAGACGCGACCGCGACTAAAACCGAAGCGGAGGCACAATG
eutA primer dn : TGTGGTCTTTAGTTTCATAAGTCGTTCCCTCAGGAAGGAAATGCGAGTGA

```
eutA primer dn rev-complement:
                  TCACTCGCATTTCCTTCCTGAGGGAACGACTTATGAAACTAAAGACCACA
                  |||||||||||||||||||||
eutA genome:    AATCACTCGCATTTCCTTCCTGAATGCGGTATTCGCCAGCGG
```

```
eutA primer dn rev-complement:
                  TCACTCGCATTTCCTTCCTGAGGGAACGACTTATGAAACTAAAGACCACA
                               |||||||||||||||||||||||||||||||||||||
eutB genome:          TCATCGATTCTTCCTGAGGGAACGACTTATGAAACTAAAGACCACA
```

The primer to construct the *eutA* Keio mutant has homology to the *eutAB* junction in *E. coli* BW25113 reference genome in the absence of the CPZ-55 cryptic prophage.

The artefact protein that arises when the *flu* mutant is constructed and cassette removed:

>BW25113_Flu - artefact
M-MIPGIRRPAVRSSTSLGSIGTSKQLQPT-
HTECDLLTEPSPLCGPGHHDRDPDRRNGSSTPHSLALTIT*

*Flu start codon – cassette scar – neighbouring ORF

Construction of the mltC mutant using the primers reported in the Keio paper:

>BW25113_2963 mltC membrane-bound lytic murein transglycosylase C 3097792:3098871 forward
ATG-AAA-AAA-TAT-CTC-GCG-CTG-GCT-TTG-ATT-GCG-CCG-TTG-CTC-ATC-
TCC-TGT-TCG-ACG-ACC-AAA-AAA-GGC-GAT-ACC-TAT-AAC-GAA-GCC-TGG-
GTC-AAA-GAT-ACC-AAC-GGT-TTT-GAT-ATT-CTG-ATG-GGG-CAA-TTT-GCC-
CAC-AAT-ATT-GAG-AAC-ATC-TGG-GGC-TTC-AAA-GAG-GTG-GTG-ATC-GCT-
GGT-CCT-AAG-GAC-TAC-GTG-AAA-TAC-ACC-GAT-CAA-TAT-CAG-ACC-CGC-
AGC-CAC-ATC-AAC-TTC-GAT-GAC-GGT-ACG-ATT-ACT-ATC-GAA-ACC-ATC-
GCC-GGG-ACA-GAA-CCT-GCC-GCG-CAT-TTG-CGC-CGG-GCA-ATT-ATC-AAA-
ACG-TTA-TTG-ATG-GGT-GAC-GAT-CCG-AGT-TCG-GTC-GAT-CTC-TAT-TCC-
GAC-GTT-GAT-GAT-ATT-ACG-ATT-TCG-AAA-GAA-CCT-TTC-CTT-TAC-GGT-
CAG-GTG-GTG-GAC-AAC-ACC-GGG-CAG-CCG-ATT-CGC-TGG-GAA-GGT-CGC-
GCA-AGC-AAC-TTC-GCG-GAT-TAT-CTG-CTG-AAA-AAC-CGT-CTG-AAG-AGC-
CGC-AGC-AAC-GGG-CTG-CGT-ATC-ATC-TAC-AGC-GTC-ACC-ATT-AAC-ATG-
GTG-CCG-AAC-CAC-CTT-GAT-AAA-CGT-GCG-CAC-AAA-TAT-CTC-GGC-ATG-
GTC-CGC-CAG-GCG-TCA-CGG-AAA-TAT-GGC-GTT-GAT-GAG-TCG-CTG-ATT-
CTG-GCA-ATT-ATG-CAG-ACC-GAA-TCT-TCC-TTT-AAC-CCG-TAT-GCG-GTC-
AGC-CGT-TCC-GAT-GCG-CTG-GGA-TTA-ATG-CAG-GTG-GTA-CAA-CAT-ACT-
GCC-GGG-AAA-GAT-GTG-TTC-CGC-TCG-CAG-GGG-AAA-TCC-GGC-ACG-CCG-
AGC-CGC-AGT-TTC-TTG-TTT-GAT-CCT-GCC-AGC-AAT-ATT-GAT-ACC-GGC-
ACC-GCG-TAT-CTG-GCG-ATG-CTG-AAC-AAT-GTT-TAT-CTC-GGC-GGA-ATT-
GAT-AAC-CCA-ACA-TCG-CGG-CGT-TAT-GCC-GTC-ATC-ACC-GCC-TAT-AAC-
GGC-GGC-GCA-GGC-AGC-GTG-CTG-CGA-GTC-TTT-TCG-AAT-GAT-AAG-ATT-
CAG-GCT-GCC-AAT-ATT-ATT-AAC-ACC-ATG-ACG-CCG-GGC-GAT-GTT-TAT-
CAG-ACG-CTG-ACG-ACC-CGC-CAT-CCC-TCT-GCG-GAA-TCT-CGC-CGT-TAT-
CTT-TAT-AAA-GTG-AAT-ACC-GCG-CAA-AAA-TCC-TAC-CGC-CGC-CGA-TAA

NNN = region deleted by kanamycin resistance cassette

Truncation of *hcaT* when the *csiE* mutant is constructed:

>hcaT MFS (major facilitator superfamily) transporter - 1970265: 1971404 MW: 41593.21
MVLQSTRWLALGYFTYFFSYGIFLPFWSVWLKGIGLTPETIGLLLGAGLVARFLGSLLIAPRVSDPSR
LISALRVLALLTLLFAVAFWAGAHVAWLMLVMIGFNLFFSPLVPLTDALANTWQKQFPLDYGKVRLWG
SVAFVIGSALTGKLVTMFDYRVILALLTLGVASMLLGFLIRPTIQPQGASRQQESTGWSAWLALVRQN
WRFLACVCLLQGAHAAYYGFSAIYWQAAGYSASAVGYLWSLGVVAEVIIFALSNKLFRRCSARDMLLI
SAICGVVRWGIMGATTALPWLIVVQILHCGTFTVCHLAAMRYIAARQGSEVIRLQAVYSAVAMGGSIA
IMTVFAGFLYQYLGHGVFWVMALVALPAMFLRPKVVPSC

*12TM domains underlined

NNN = region deleted by kanamycin resistance cassette

NNN = TM domain (x12)

Primers for amplification of the cassette-gDNA junction:
K1F 5′-TCGCCTTCTTGACGAGTTCTTCTAATAAGG-3′
A1R 5′-GACTGGAGTTCAGACGTGTGCTCTTCCGATC-3′
K2F 5′-
AATGATACGGCGACCACCGAGATCTACACTCTTTCCCTACACGACGCTCTTCCGATCTNNNNNNAAA
GTATAGGAACTTCGAAGCAGCT-3′
*Underlined section has homology to the kanamycin resistance cassette
NNN = barcode
A2R – NEBNext Illumina Index primers were used

Supplementary Figures

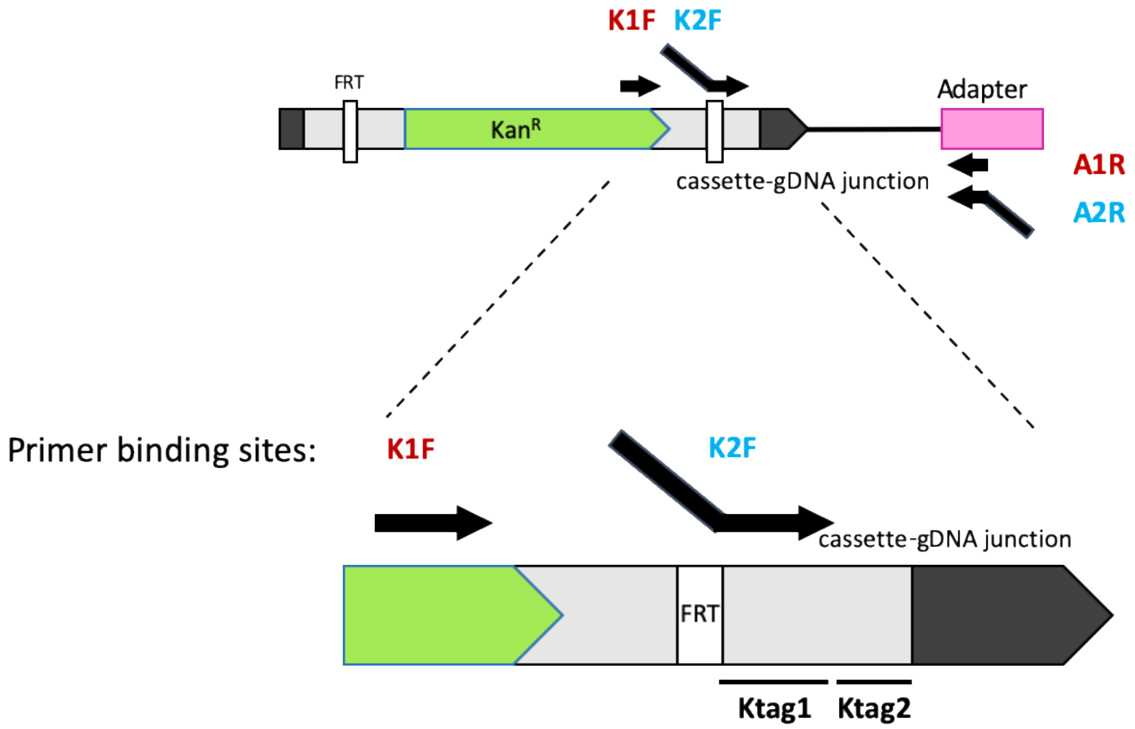

FIG S1 Primer binding sites for amplification of the cassette-gDNA junctions for sequencing Following DNA fragmentation, the cassette-gDNA junctions are enriched by PCR using primer pair K1F and A1R. The sample is then prepared for sequencing by PCR using the semi-nested primer pair K2F and the NEBNext® Multiplex Oligos for Illumina, denoted here as 'A2R'. These primers introduce the necessary barcodes and flow cell-binding adapters required for Illumina sequencing. During processing of FASTQ data, the kanamycin resistance cassette is recognized in two pattern matching steps that correspond with Ktag1, allowing for 3 mismatches, and Ktag2, allowing for 1 nucleotide mismatch. The Ktag1 sequence corresponds with the terminal 25 nucleotides of the K2F primer. FRT = Flp recognition target; gDNA = genomic DNA.

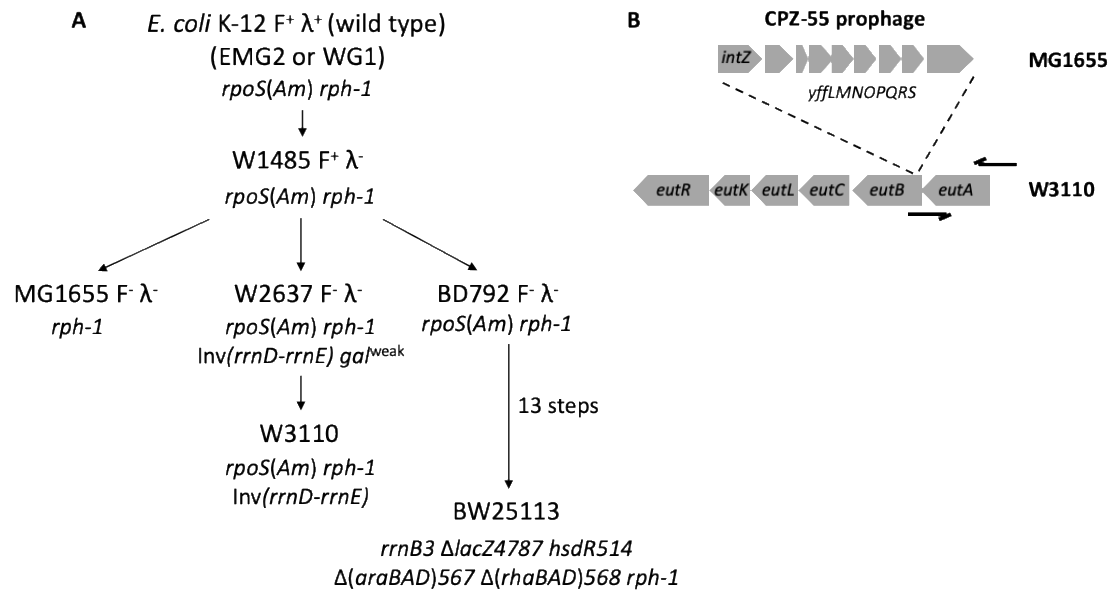

FIG S2 The relationship between *E. coli* K-12 strains BW25113, MG1655 and W3110
(A) Figure adapted from Bachmann [1], Baba *et al*. and Hayashi *et al.* [2,3]. (B) The *eut* operon of MG1655 and W3110 and the primer binding sites for the primers used to construct the *eutA* mutant.

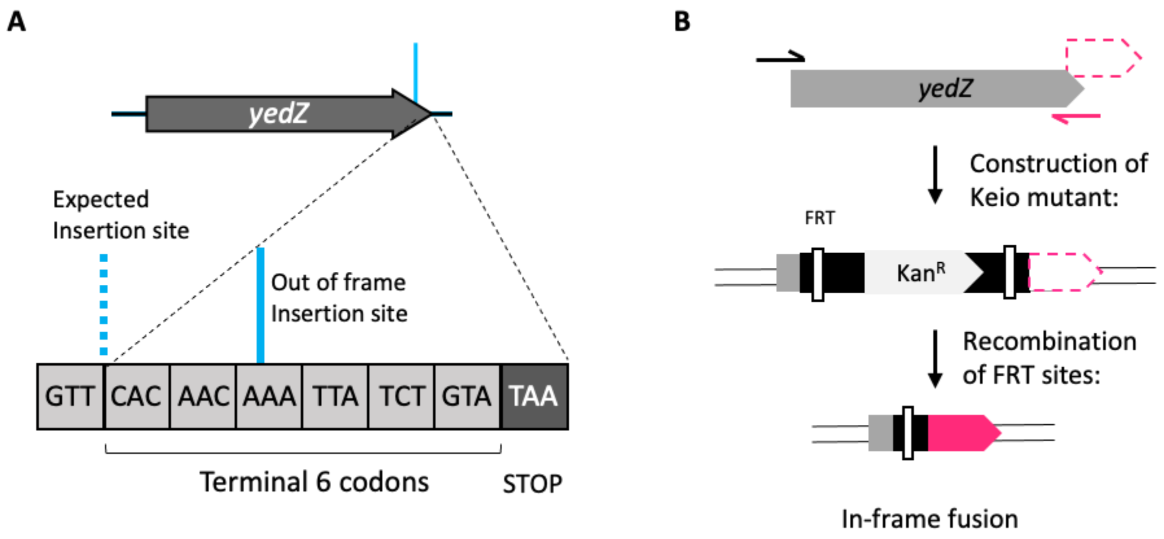

FIG S3 Identification of an out-of-frame insertion within *yedZ*

(A) Insertion of the kanamycin resistance cassette in the *yedZ* mutant. (B) Excising the kanamycin resistance cassette in the *yedZ* mutant will result in construction of an artefact coding sequence.

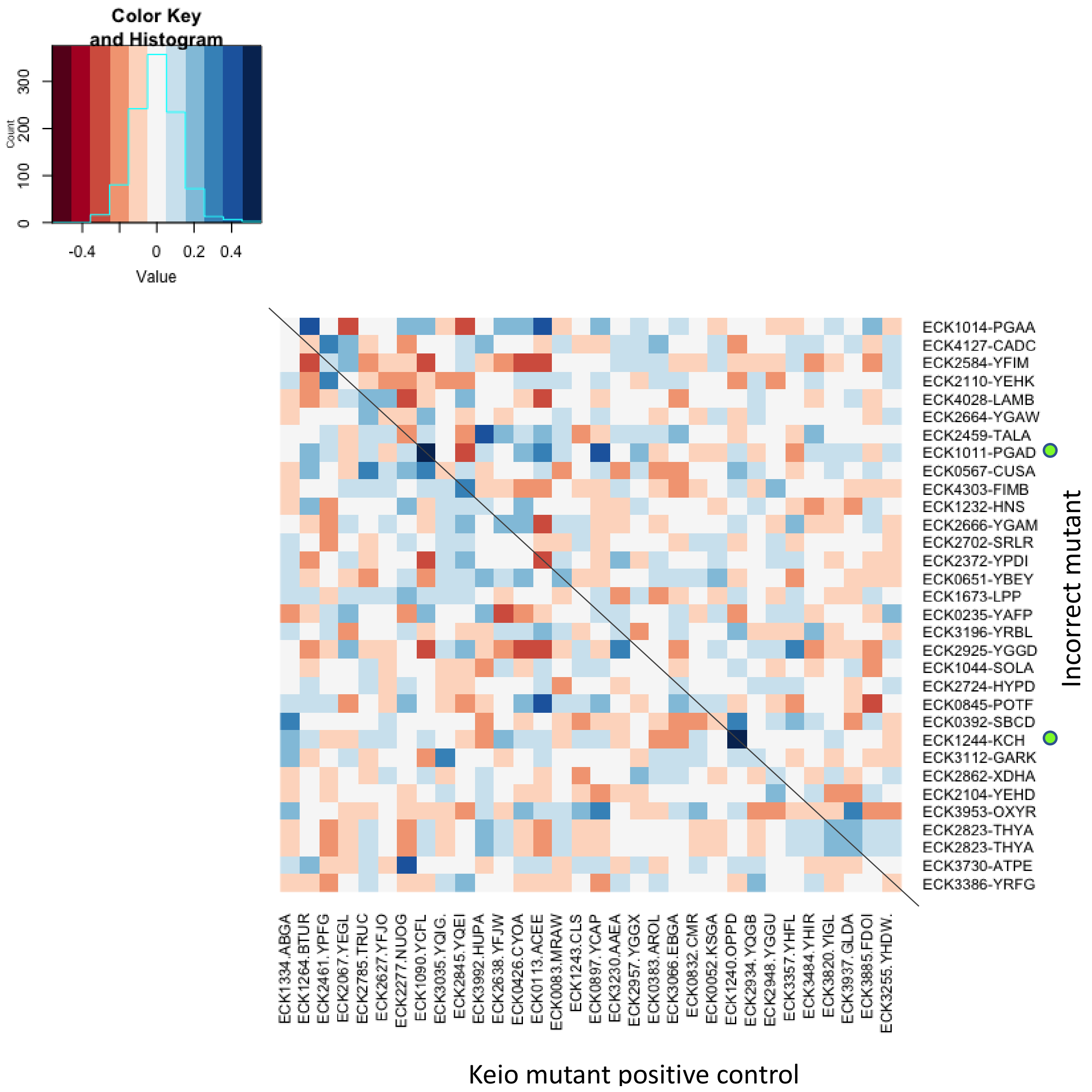

FIG S4 Comparison of the phenotypic profiles of Keio mutants with an incorrectly positioned kanamycin resistance cassette

Comparison of phenotypic correlation scores, taken from Nichols *et al.* [4]. Mutants with an incorrectly located kanamycin resistance cassette (y-axis) were compared to the corresponding mutant (x-axis). Mutants with a negative phenotypic correlation are shown in red, mutants with a positive phenotypic correlation are shown in blue.

Supplementary Tables

TABLE S1 Mutants that failed to grow

ECK	*Name*	b	JW	Plate	Column	Row
ECK2278	*nuoF*	b2284	JW2279	3	4	G
ECK0355	*yaiO*	b0358	JW0349	15	4	A
ECK2289	*yfbV*	b2295	JW2292	18	4	D
ECK2008	*plaP*	b2014	JW5330	70	1	E

Supplementary Datasets

DATASET S1 Keio mutant gene-disruption status

DATASET S2 Identification of the kanamycin resistant cassette insertion site by PCR

References

1. Bachmann BJ. Pedigrees of some mutant strains of *Escherichia coli* K-12. Bacteriol Rev. 1972 Dec;36(4):525–57.
2. Baba T, Ara T, Hasegawa M, Takai Y, Okumura Y, Baba M, Datsenko K a, Tomita M, Wanner BL, Mori H. Construction of *Escherichia coli* K-12 in-frame, single-gene knockout mutants: the Keio collection. Mol Syst Biol. 2006;2:2006.0008.
3. Hayashi K, Morooka N, Yamamoto Y, Fujita K, Isono K, Choi S, Ohtsubo E, Baba T, Wanner BL, Mori H, Horiuchi T. Highly accurate genome sequences of *Escherichia coli* K-12 strains MG1655 and W3110. Mol Syst Biol. 2006;2:2006.0007.
4. Nichols RJ, Sen S, Choo YJ, Beltrao P, Zietek M, Chaba R, Lee S, Kazmierczak KM, Lee KJ, Wong A, Shales M, Lovett S, Winkler ME, Krogan NJ, Typas A, Gross CA. Phenotypic landscape of a bacterial cell. Cell. 2011 Jan 7;144(1):143–56.
